# Whole-Genome Sequences and Annotation of the Opportunistic Pathogen *Candida albicans* Strain SC5314 Grown under Two Different Environmental Conditions

**DOI:** 10.1128/genomeA.01475-17

**Published:** 2018-02-01

**Authors:** Thais Fernanda Bartelli, Danielle do Carmo Ferreira Bruno, Marcelo R. S. Briones

**Affiliations:** a Laboratory of Evolutionary Genomics and Biocomplexity, Department of Microbiology, Immunology and Parasitology, Universidade Federal de São Paulo, São Paulo, SP, Brazil; b Department of Health Informatics, Universidade Federal de São Paulo, São Paulo, SP, Brazil

## Abstract

The genetic variability of the opportunistic pathogen *Candida albicans* is an important adaptive mechanism. Here, we present the whole-genome sequences of the *C. albicans* SC5314 strain under two different growth conditions, providing useful information for comparative genomic studies and further intraspecific analysis.

## GENOME ANNOUNCEMENT

The fungus *Candida albicans* is ubiquitously found in the human body and successfully colonizes diverse niches, such as skin and urogenital and gastrointestinal tracts, including internal organs, after tissue invasion and bloodstream dissemination (reviewed in references 1, 2, and 3). Although part of the human microbiota, *C. albicans* causes severe mucosal and bloodstream opportunistic infections in immunosuppressed hosts, with nearly 400,000 nosocomial cases worldwide with 46 to 75% mortality rates (4).

We used the *C. albicans* strain SC5314, kindly provided to our laboratory by A. Mitchell in the mid-1990s. This strain is considered a reference strain and was isolated in 1984 from a candidemia patient (5). Since then, samples of this strain have been distributed to many laboratories and used in studies worldwide. The genome of one of these samples was previously sequenced by Muzzey and collaborators (6) using next-generation sequencing technologies.

Our SC5314 (named SC5314-P0) yeast cells were grown on yeast extract-peptone-dextrose (YPD) plates (1% wt/vol yeast extract; 2% wt/vol peptone, 2% wt/vol dextrose, and 2% wt/vol agar), and a single colony was used for overnight growth on YPD broth at 28°C and 150 rpm. Total yeast DNA was extracted from samples as described previously (7), and the complete sequencing of mitochondrial and nuclear genomes was carried out by using the Illumina MiSeq 2 × 300-bp method in paired-end mode. The libraries were prepared with a TruSeq DNA v2 Illumina kit according to the manufacturer’s technical specifications. FastQC v.0.11.4 software (8) was used to evaluate sequencing quality. Trimming was performed with the CLC Genomics Workbench v.7.5.1 (Qiagen) software with a quality score limit of 0.005 and removal of 45 bp and 20 bp from the 5′ and 3′ ends, respectively, and reads smaller than 25 bp were discarded. Once the quality filters were approved, reads were mapped to the assembly 22 of the reference strain SC5314 (A22-s07-m01-r18) (available at http://www.candidagenome.org/download/sequence/C_albicans_SC5314/Assembly22/archive/). Duplicated reads were removed after mapping and local realignment were carried out with the Guided Realignment tool (with “force realignment to guidance variants” selected) implemented in CLC Genomics Workbench v.7.5.1 (Qiagen). Genome annotation was performed with Annotate with the GFF file tool available on the same software using the corresponding GFF file (version A22-S05-M04-r02_features_with_chromosome_sequences.gff). The fraction of the SC5314 genome sequenced was 0.99 to 1, with an average coverage ranging from 20.85× to 40×, depending on the yeast nuclear chromosome, while the mitochondrial DNA (mtDNA) was sequenced with an average coverage of 11,236×.

We also sequenced the whole genome of the strain SC5314 after 12 weeks of continuous growth under an oxygen level of 5 to 15% (hypoxia), yeast extract-peptone-glycerol (YPG) broth (1% wt/vol yeast extract; 2% wt/vol peptone, 2% wt/vol glycerol) at 37°C, named SC5314-GTH12 (for glycerol, thirty-seven °C, hypoxia, 12 weeks). The consensus sequence of the SC5314 strain was extracted and used as a reference for the mapping of sample SC5314-GTH12. Mapping of paired-end reads, after removal of duplicated reads and local realignment, resulted in an average coverage of 15.9× to 26.8× for the yeast nuclear chromosomes and 1,920× for its mitochondrial genome. Satisfactory fractions of chromosome sequences were obtained, ranging from 0.98 to 1.00.

### Accession number(s).

*C. albicans* sequences obtained in this study have been deposited in GenBank (https://www.ncbi.nlm.nih.gov/nucleotide/). Accession numbers are designated separately for both copies of the *C. albicans* diploid genomes sequenced (A and B). The GenBank accession numbers for the sample of strain SC5314-P0 are CP025150 (chromosome 1A), CP025151 (chromosome 2A), CP025152 (chromosome 3A), CP025153 (chromosome 4A), CP025154 (chromosome 5A), CP025155 (chromosome 6A), CP025156 (chromosome 7A), CP025157 (chromosome RA), CP025158 (chromosome 1B), CP025159 (chromosome 2B), CP025160 (chromosome 3B), CP025161 (chromosome 4B), CP025162 (chromosome 5B), CP025163 (chromosome 6B), CP025164 (chromosome 7B), CP025165 (chromosome RB), and CP025166 (mitochondrial DNA). The sample SC5314-GTH12 accession numbers are CP025167 (chromosome 1A), CP025168 (chromosome 2A), CP025169 (chromosome 3A), CP025170 (chromosome 4A), CP025171 (chromosome 5A), CP025172 (chromosome 6A), CP025173 (chromosome 7A), CP025174 (chromosome RA), CP025175 (chromosome 1B), CP025176 (chromosome 2B), CP025177 (chromosome 3B), CP025178 (chromosome 4B), CP025179 (chromosome 5B), CP025180 (chromosome 6B), CP025181 (chromosome 7B), CP025182 (chromosome RB), and CP025183 (mitochondrial DNA).

## References

[1] Brock M 2009 Fungal metabolism in host niches. Curr Opin Microbiol 12:371–376. doi:10.1016/j.mib.2009.05.004. 19535285

[2] Huffnagle GB, Noverr MC 2013 The emerging world of the fungal microbiome. Trends Microbiol 21:334–341. doi:10.1016/j.tim.2013.04.002. 23685069 PMC3708484

[3] Underhill DM, Iliev ID 2014 The mycobiota: interactions between commensal fungi and the host immune system. Nat Rev Immunol 14:405–416. doi:10.1038/nri3684. 24854590 PMC4332855

[4] Brown GD, Denning DW, Gow NAR, Levitz SM, Netea MG, White TC 2012 Hidden killers: human fungal infections. Sci Transl Med 4:165rv13. doi:10.1126/scitranslmed.3004404. 23253612

[5] Gillum AM, Tsay EYH, Kirsch DR 1984 Isolation of the *Candida albicans* gene for orotidine-5′-phosphate decarboxylase by complementation of *S. cerevisiae* ura3 and *E. coli* pyrF mutations. Mol Genet 198:179–182. doi:10.1007/BF00328721. 6394964

[6] Muzzey D, Schwartz K, Weissman JS, Sherlock G 2013 Assembly of a phased diploid *Candida albicans* genome facilitates allele-specific measurements and provides a simple model for repeat and indel structure. Genome Biol 14:R97. doi:10.1186/gb-2013-14-9-r97. 24025428 PMC4054093

[7] Wach A, Pick H, Philippsen P 1994 Procedures for isolating yeast DNA for different purposes. *In* Johnston JR (ed), Molecular genetics of yeast. IRL Press at Oxford University Press, Oxford, United Kingdom.

[8] Andrews S 2010 FastQC: a quality control tool for high throughput sequence data. http://www.bioinformatics.babraham.ac.uk/projects/fastqc.

